# BRD4 Phosphorylation Regulates the Structure of Chromatin Nanodomains

**DOI:** 10.3390/cells15020118

**Published:** 2026-01-09

**Authors:** Clayton Seitz, Donghong Fu, Mengyuan Liu, Hailan Ma, Jing Liu

**Affiliations:** 1 Department of Physics, Indiana University, Indianapolis, IN 46202, USA; fu425@purdue.edu (D.F.); mengyuanliu0524@163.com (M.L.); mahai@iu.edu (H.M.); 2 Department of Physics and Astronomy, Purdue University, West Lafayette, IN 47907, USA; 3 Purdue Institute for Cancer Research, Purdue University, West Lafayette, IN 47907, USA; 4 Melvin and Bren Simon Comprehensive Cancer Center, Indiana University, Indianapolis, IN 46202, USA

**Keywords:** BRD4, condensation, chromatin, super resolution

## Abstract

The interplay between chromatin structure and phase-separating proteins is an emerging topic in cell biology with implications for understanding disease states. Here, we investigate the functional relationship between bromodomain protein 4 (BRD4) and chromatin architecture. By combining molecular dynamics simulations with live-cell imaging, we demonstrate that BRD4, when mutated at specific N-terminus sites, significantly impacts the organization and dynamics of chromatin nanodomains, known as nucleosome clutches. Our findings reveal that a constitutively phosphorylated mutant of BRD4 condenses nucleosome clutches, while treatment with (+)-JQ1 increases the diffusion dynamics of single nucleosomes and decondenses nucleosome clutches. Simultaneously, we demonstrate that BRD4 mutations can alter localization of BRD4 to chromatin as well as modify single nucleosome dynamics. These results suggest that both chromatin binding and phase separation of BRD4 could co-regulate the nanoscale chromatin architecture and the chromatin microenvironment. Our observations shed light on the nuanced regulation of chromatin structure by BRD4, offering insights into its role in maintaining the nuclear architecture and transcriptional activity.

## 1. Introduction

The cell nucleus is a densely packed environment, with chromatin comprising a dominant component. Emerging research supported by advanced imaging and sequencing methods has revealed that chromatin is dynamic and highly compartmentalized [1,2]. The compartmentalization of chromatin with other intranuclear components is therefore an efficient strategy to ensure precise spatial and temporal coordination of complex dynamics towards the regulation of intracellular activities and cellular functions. On one hand, nanoscale chromatin movement (chromatin motion) modulates the interaction of DNA with regulatory molecules (chromatin accessibility), thereby influencing global gene expression patterns. The structure of chromatin nanodomains, which are the assembly of nucleosomes described as nucleosome clutches (NCs), was found to be relevant to epigenetic modifications [3,4,5]. Meanwhile, a growing number of phase-separated nuclear bodies, including transcriptional condensates [6,7,8,9,10,11,12], nuclear speckles [13,14,15,16,17], and DNA damage repair foci [18,19,20,21,22], have been identified. However, the interplay of phase-separated condensates with the underlying chromatin structure remains poorly understood.

Transcriptional condensates are a suitable model to study the kinetic and thermodynamic contributions of chromatin substrate binding, as the ability of transcriptional activators to both condense and bind chromatin is well established [6,23,24,25,26]. For example, BRD4 protein is a well-studied transcriptional activator that undergoes phase separation [27], localizes to acetylated chromatin sites [28], recruits pTEF-b, and initiates transcription of key genes involved in signal response, immunity, and oncogenesis [29]. The BRD4 long isoform is characterized by structured N-terminal tandem acetyl-lysine binding bromodomains and an extra-terminal domain, connected by intrinsically disordered regions [30].

Recent evidence suggests a phosphorylation-dependent binding mechanism of BRD4 to acetylated chromatin [31,32]. However, the binding kinetics of BRD4 proteins with chromatin cannot be directly transferred to studying the interplay of transcriptional condensates with the underlying chromatin structure, and the mechanistic role of chromatin dynamics in transcriptional regulation remains unclear. Therefore, we speculated that BRD4 phosphorylation can modulate a multivalent binding interaction between the transcriptional condensate with NCs, making BRD4 phosphorylation necessary for the maintenance of chromatin structure and dynamics.

## 2. Materials and Methods

### 2.1. Cell Lines, Cell Culture Conditions, and Transfection

HeLa cells were cultured in DMEM supplemented with 10% fetal bovine serum (Gibco) at 37 °C and 5% CO_2_ in a humidified incubator. Cultures were tested routinely for mycoplasma contamination; all tests were negative. For imaging experiments, cells were seeded in a 35 mm FluoroDish (WPI), and transfected using Lipofectamine 3000 (ThermoFisher #L3000008, Waltham, MA, USA), with various plasmids from Addgene, Watertown, MA, USA (pBREBAC-H2B-Halo, Addgene #91564; pcDNA5-Flag-BRD4-7A, Addgene #90006; pcDNA5-Flag-BRD4-7D; Addgene #90007; pcDNA5-Flag-BRD4-BD, Addgene #90005; pcDNA5-Flag-BRD4-WT, Addgene #90331; and GFP-BRD4, Addgene #65378). Co-transfection of H2B-Halo and FLAG-tagged BRD4 mutants was optimized such that cells which expressed H2B-Halo also expressed the BRD4 mutant, making H2B-Halo expression an indicator of expression/no expression of FLAG-tagged BRD4. FLAG-tagged BRD4 mutants were homogeneously expressed across cells in which they were expressed. Optimization was carried out iteratively by simultaneous imaging of H2B-Halo and immunofluorescence of FLAG-tagged BRD4 in fixed cells.

### 2.2. Plasmid DNA Purification

Plasmids were transformed in *E. coli* at 4 °C and selected using an antibiotic agar plate. A single colony from the plate was selected and placed into sterile antibiotic LB Broth, followed by incubation with shaking at 37 °C for 12 h. After amplification, DNA was purified using a Miniprep kit (Promega, Madison, WI, USA). Following extraction, the concentration and purity were measured using the Nanodrop 2000 Software on Microsoft Windows 7. Plasmids were stored with optimal concentration and purity at −20 °C. All experiments involving (+)-JQ1 (MedChemExpress, Monmouth Junction, NJ, USA) were carried out by exposing living HeLa cells to a 1 μM concentration for 8 h.

### 2.3. Single-Molecule Tracking of Single Nucleosomes in Live Hela Cells

For single-molecule tracking experiments to capture the dynamics of individual single nucleosomes in 2D, H2B-Halotag HeLa cells were incubated with 3 pM JF646 HaloTag ligand for 1 h. The same single-molecule imaging system was used, but the laser illumination was set to 10 mW, and 100 frames were captured at 10 frames per second. Three replicates were conducted for each group, and 15–20 cells were collected per group. Nucleosomes were localized as in super-resolution experiments and tracked using TrackPy Python software (Version 0.6.1) [33]. Trajectories lasting less than 80 frames were removed from further analysis. The individual mean squared displacement (MSD) was computed as follows:

$$\langle {\Delta r}^{2}(\tau )\rangle \;=\;\frac{1}{\left|S_{\tau }\right|}\sum_{\Delta r\in S_{\tau }}{\left(\Delta r\right)}^{2}$$

where Δr is the 2D displacement vector and $S_{\tau }$ is the set of all displacements in a time interval τ. The diffusion coefficient for both simulations as well as experimental data was computed by linear regression of the formula:

$$log\;\langle {\Delta r}^{2}(\tau )\rangle \;=\;log\;4D\;+\;\alpha \;log\;\tau$$

The symbol D represents the diffusion coefficient, α is the anomalous exponent, and τ is the lag time.

### 2.4. Super-Resolution Imaging of Nucleosome Nanodomains in Living Cells

After transient transfection, H2B-Halotag HeLa cells were incubated with 3 pM JF646 HaloTag ligand overnight. Cells were imaged in 2D, using a dSTORM photoswitching buffer containing phenol-red free cell culture medium, 100 mM MEA, 50 μg/mL Glucose Oxidase, 3.4 mg/mL Catalase (Sigma, St. Louis, MO, USA), and 10% Glucose. Buffer pH was adjusted to ~8 using HCl. Movies were collected using a custom Olympus IX83 microscope body equipped with an Olympus 60X 1.25NA oil-immersion objective (Evident Scientific, Tokyo, Japan). During imaging, cells were maintained at 37 °C and 5% CO_2_ in a stage top incubator (Tokai Hit, Fujinomiya City, Japan). Images were projected onto an ORCA-Fusion sCMOS camera (Hamamatsu, Hamamatsu, Japan), and 2000 frames were captured at 100 fps. The microscope was controlled using Micromanager software (Version 2.0.0). HaloTag-JF646 molecules were imaged using oblique illumination with a 640 nm laser (Excelitas, Pittsburgh, PA, USA) held at 20 mW, as measured at the back focal plane of the objective. Super-resolution reconstructions were obtained using the ThunderSTORM ImageJ plugin (Version 1.54) [34]. Background signal was subtracted using a rolling ball filter with radius of 10 pixels. Spots were fit using an integrated Gaussian point spread function model with maximum likelihood estimation [35,36]. Three replicates were conducted for each group, and statistical analyses were performed with N = 15–20 cells per group.

After precise positions of the fluorophores were obtained, Besag’s L-function L(r) was used to analyze the clustering. The L-function is a transformation of Ripley’s K-function. The K-function is designed such that K(r) is the number of localizations within a radius r of a randomly chosen localization. Importantly, in the case of complete spatial randomness, L(r)=r. Thus, in general, to quantify the degree of clustering, one uses L(r)−r, which measures the deviation of a point pattern from complete spatial randomness.

### 2.5. Colocalization of BRD4 Mutants with Nucleosome Clutches

H2B-Halo was transiently transfected into HeLa cells, which were then incubated with JF646 HaloTag ligand. Cells were fixed with Formaldehyde in 1x PBS at 37 °C in an incubator for 20 min, and then permeabilized with 0.3% (*v*/*v*) Triton-X100 (Sigma-Aldrich, St. Louis, MO, USA) in PBS and blocked for 1 h in 5% (*w*/*v*) nonfat dry milk (Bio-Rad, Hercules, CA, USA) at 4 °C. Cells were incubated overnight at 4 °C using anti-FLAG primary antibodies (Sigma-Aldrich, #F1804, 1:1000). Cells were incubated with secondary antibodies (Cell Signaling Anti-Mouse IgG-Alexa488, #4408S, 1:1000, Danvers, MA, USA) for 45 min at room temperature before imaging.

We colocalized FLAG-tagged BRD4 mutants with nucleosome in 2D by simultaneous FLAG immunofluorescence with imaging of sparsely labeled H2B-JF646. Puncta were detected in both channels using the Laplacian of Gaussian (LoG) detection algorithm to generate a multi-type point pattern. We then computed a colocalization ratio R, per cell:

$$R=\frac{\langle d_{H2B\to FLAG}\rangle }{\langle d_{FLAG\to FLAG}\rangle }$$

where $\langle d_{H2B\to FLAG}\rangle$ represents the averaged distance from an H2B puncta to its nearest neighboring FLAG puncta. To address density variations in FLAG over the cell population, we normalized this quantity to the averaged FLAG-FLAG nearest-neighbor distance $\langle d_{FLAG\to FLAG}\rangle .$ Three replicates were conducted for each group, and statistical analysis was performed with N = 40–60 cells per group.

### 2.6. Immunoblotting

Cells were washed, and lysis buffer added (RIPA buffer:PMSF:protease inhibitor cocktail:orthovanadate = 100:1:2:1). Cells were then scraped and sonicated for 15 s using an ultrasonic homogenizer (Qsonica, Q500, Newtown, CT, USA). Lysate was centrifuged at high speed (13,200 RPM) for 15 min at 4 °C to pellet the cellular debris. Total protein concentration was determined by the BCA Protein Assay Kit (Thermo Fisher). For electrophoresis, protein samples were prepared according to a protein-4x loading buffer (containing DTT) ratio of 3:1; 4x loading buffer containing DTT was diluted with 3 aliquots of protein sample. The sample was mixed and vortexed, then heated at 95 °C for 5 min, followed by vortexing and centrifuging. After running the gel, it was removed from the cassette and assembled inside the Trans-Blot Turbo Transfer System cassette. Transfer was run at 2.5 A and 25 V for 7 min. The sample was then blocked for at least 1 h using 5% skim milk blocking solution prepared with PBS at RT. Primary FLAG antibody was diluted in PBST with 3% skim milk (1:500) and incubated at 4 °C overnight. The secondary antibody (Licor Anti-Mouse IgG-IRDye 800 CW, Lincoln, NE, USA) was diluted in PBST with 3% skim milk (1:5000) and placed on a rocker and incubated in the dark at RT for 45 min. Western blots on Nitrocellulose membranes were scanned using the Odyssey fluorescence scanning system software. N = 2 biological replicates were conducted for this experiment.

## 3. Results

### 3.1. Colocalization of BRD4 Mutants with Nucleosomes

**Figure 1:**
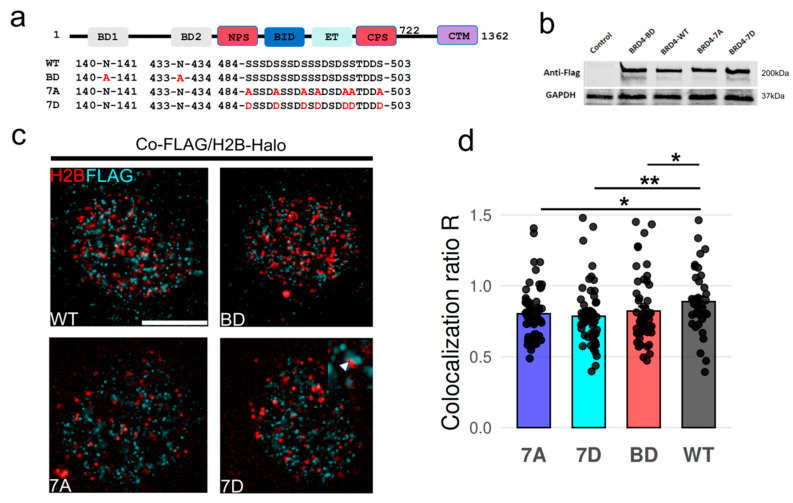
BRD4 mutants colocalize with nucleosome nanodomains. (**a**) Schematic of the BRD4 protein sequence and mutations shown in red. (**b**) Anti-FLAG Western blot of bulk expression of BRD4 mutants. (**c**) Combined immunofluorescence of FLAG-tagged BRD4 mutants with JF646-tagged H2B-Halo. Scale bar 5 μm. (inset in 7D) White arrowhead highlights proximal FLAG-tagged BRD4 and H2B puncta. (**d**) Bar plots of the colocalization ratio calculated per cell over N = 40–60 cells for each mutant. Statistical significance determined by Mann–Whitney test for each mutant relative to wild-type (WT) * *p* < 0.05, ** *p* < 0.01. N = 3 biological replicates were performed for each group.

To address the role of BRD4 binding and phase separation on chromatin structure, we expressed FLAG-tagged BRD4 mutants in HeLa cells and measured their effects on chromatin organization. Perhaps the most fundamental of BRD4 functions is the ability to bind to acetylated chromatin through bromodomain 1 (BD1) and bromodomain 2 (BD2) that are in tandem within the N-terminal part of the protein. BRD4 inhibitors such as (+)-JQ1 competitively bind to the acetyl-binding pocket of BRD4, displacing BRD4 from chromatin [30]. It is also known that BRD4 association with acetylated chromatin is enhanced by casein kinase II (CK2)-mediated phosphorylation of seven N-terminus phosphorylation sites (NPS), followed by BRD4 dimerization [31] or intramolecular rearrangement [32]. Therefore, we expressed a constitutively phosphorylated BRD4 mutant with seven aspartate substitutions in the NPS region (7D mutant), a constitutively unphosphorylated mutant with seven alanine substitutions in the NPS region (7A mutant), and bromodomain-deactivated (BD mutant) (Figure 1a). We found similar bulk expression levels of all mutants (Figure 1b) as well as similar nuclear expression levels of mutants as determined by imaging H2B-Halo and FLAG simultaneously. We found no significant difference in the overall density of FLAG puncta per nucleus, Ripley’s K-function of FLAG puncta surrounding H2B, or intensity/size of FLAG puncta (Supplementary Figures S1–S3). Colocalization analysis of BRD4 mutants with nucleosomes in 2D using the nearest-neighbor distance distribution showed an obvious colocalization of these mutants with nucleosomes. The 7D mutant showed the closest proximity to nucleosomes relative to wild type, followed by BD and 7A mutants (Figure 1d). This result is consistent with the known dependence of BRD4 chromatin binding on phosphorylation state. It should be noted that there are multiple factors involved in BRD4 recruitment to the chromatin [27], and tested mutations may not impair all mechanisms simultaneously. Therefore, we did not observe a complete loss of BRD4 recruitment to nucleosomes with respect to the wild type.

### 3.2. Phase-Separated BRD4 Condensates Regulate Chromatin Structure and Dynamics

**Figure 2:**
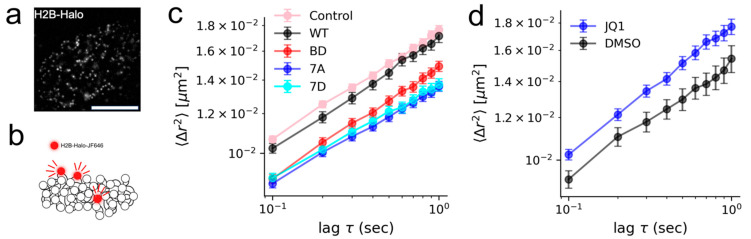
BRD4 mutants reduce single nucleosome dynamics in living cells. (**a**,**b**) Sparse labeling of single nucleosomes in a living HeLa cell (scale bar 5 μm). (**c**) Average mean squared displacement (MSD) of single nucleosomes in control, wild-type BRD4, and mutated BRD4. Error bars represent the standard error of the mean. (**d**) Average MSD of single nucleosomes after 8 h exposure to 1 μM (+)-JQ1 in DMSO and DMSO only; error bars represent the standard error of the mean. Approximately N = 15–20 cells were used in each condition for analysis. N = 3 biological replicates were performed for each group.

**Figure 3:**
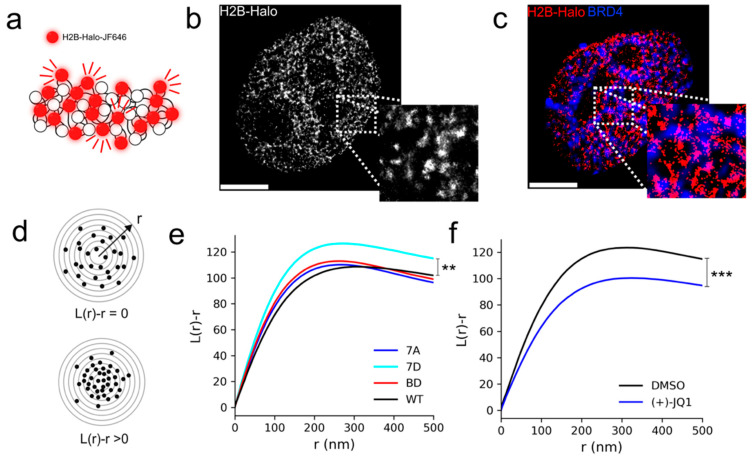
Expression of constitutively phosphorylated BRD4 compacts nucleosome nanodomains. (**a**,**b**) Direct stochastic optical reconstruction microscopy (dSTORM) imaging strategy for single nucleosomes and an example super-resolution image. (**c**) Two-color image of super-resolved H2B-JF646 with diffraction-limited GFP-tagged BRD4. (**d**) Quantification of degree of clustering using the L-function. (**e**) L-function for BRD4 mutants. (**f**) L-function after 8 h exposure to 1 μM (+)-JQ1 in DMSO and DMSO only. All scale bars 3 μm. ** *p* < 0.01, *** *p* < 0.001. Approximately N = 20–30 cells were used in each condition for analysis. N = 3 biological replicates were performed for each group.

To assess the functional role of BRD4 in maintaining the nucleosome environment, we interrogated the dynamics of chromatin, as well as its structure, in the presence of BRD4 mutants in live Hela cells. Histone H2B was tagged with HaloTag [37] (H2B-Halo), to which a fluorescent ligand, Janelia Fluor 646 (JF646), can bind specifically in a living cell. Low concentrations of JF646 were used to obtain sparse labeling of nucleosomes for single-nucleosome imaging (Figure 2a,b). JF646-labeled nucleosomes in HeLa cells were recorded in 2D at 10 fps (∼200 frames, 20 s total), and a reduced diffusion coefficient was measured in cells expressing 7A, 7D, and BD mutants, with respect to cells expressing the wild-type protein (Figure 2c). Meanwhile, HeLa cells exposed to (+)-JQ1 in DMSO for 8 h showed an increase in nucleosome dynamics with respect to DMSO alone (Figure 2d). We then conducted super-resolution imaging of chromatin using 2D direct stochastic optical reconstruction microscopy (dSTORM) by promoting JF646 fluorescence intermittency with a cysteamine buffer (Figure 3a,b). JF646 is known to exhibit a transient fluorescent state lasting tens to hundreds of milliseconds and stable dark state lasting hundreds of milliseconds to seconds.

Two-color imaging of H2B-Halo-JF646 and GFP-tagged BRD4 showed that BRD4 and NCs form complementary biomolecular condensates in the nucleus, consistent with current models of a BRD4 chromatin reading mechanism (Figure 3c). Ensemble averages of Besag’s L-function (Figure 3d) showed an increase in nucleosome clustering in cells expressing the 7D BRD4 mutant, while all other groups were consistently indistinguishable from WT cells (Figure 3e). In addition to that, HeLa cells exposed to (+)-JQ1 in DMSO for 8 h showed a reduction in nucleosome clustering with respect to DMSO alone (Figure 3f).

### 3.3. Heteropolymer Model to Simulate the Interplay Between Condensates and Chromatin

**Figure 4:**
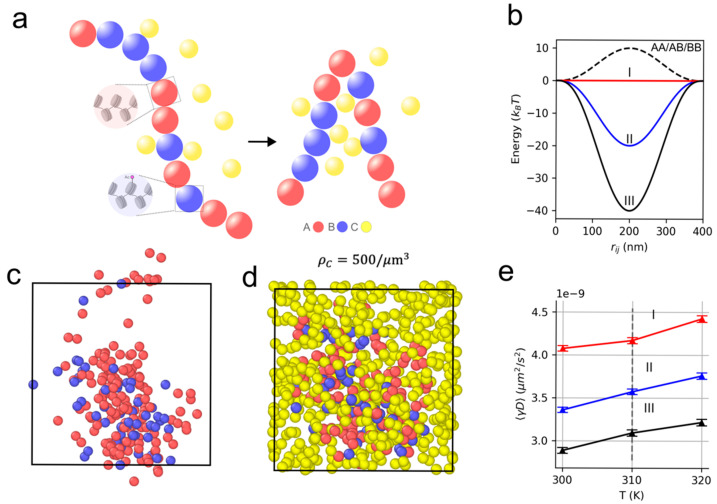
Multivalent chromatin binders reduce diffusion of chromatin beads. (**a**) Heteropolymer model of chromatin consisting of A-type, B-type, and C-type beads. (**b**) Interaction potentials $U_{BC}(r_{ij})$ of multivalent chromatin binders with B-type chromatin beads. (**c**,**d**) Examples of free heteropolymer and heteropolymer with a number density of C-type beads of $\rho =500/\mu m^{3}$ in a 10 μm periodic box. (**e**) Scaled diffusion coefficient D for various chromatin binding energies of C-type beads, averaged over 10 independent simulations, with burn-in discarded.

To interpret our experimental findings, we adopted a heteropolymer chromatin model [38] to capture the interaction of chromatin with multivalent BRD4-like binders (Figure 4a). The heteropolymer consisted of a coarse-grained bead-and-spring chain composed of $N_{b}=200$ beads, connected by harmonic bonds with equilibrium length $r_{0}$, whose energy is defined as

$$U_{AB}(r_{ij})=\frac{\kappa }{2}(|r_{ij}|-r_{0}{)}^{2}$$

where $r_{ij}$ is a vector connecting the center of a bead of type i to a bead of type j and i,j ϵ (A,B). In all simulations, we assumed $\kappa =90k_{B}T/{r_{0}}^{2}$, where $k_{B}$ is Boltzmann’s constant and $r_{0}=200\;nm$. Random beads in the chain were selected to represent locally unacetylated (A-type beads) and acetylated chromatin (B-type beads). B-type beads undergo multivalent interactions with a third group of C-type beads, which can promote crosslinking of the polymer. We assumed a Bernoulli probability of p=0.3 for any given bead to be in an acetylated-like state. Interaction of multivalent chromatin binders with chromatin beads is then mediated by the following potential [38]:

$$U_{BC}(r_{ij})=\varepsilon {\left(1-{\left(\frac{\left|r_{ij}\right|}{R_{0}}\right)}^{2}\right)}^{3}$$

where $R_{0}=200\;nm$. The potential $U_{BC}\;$ is considered over a domain $0\leq |r_{ij}|\leq 2R_{0}.\;$ In all simulations, 10 replicates were run for each condition tested. A- and B-type beads within the chromatin polymer had repulsive interactions with $\varepsilon \;=\;+10k_{B}T$. Binding energy of the acetylated beads with binders was varied with $\varepsilon_{I}\;=\;0k_{B}T,\varepsilon_{II}\;=\;-20k_{B}T,\varepsilon_{III}\;=\;-40k_{B}T$ (Figure 4b). The dynamics of chromatin chains were approximated by Brownian dynamics within a cubic box with side length of 10 μm and periodic boundary conditions. Brownian dynamics follow the stochastic differential equation:

$$\dot{r}\;=\;\gamma^{-1}\nabla U(r)\;+\sqrt{2k_{B}T}\gamma^{-1/2}\xi (t)$$

where γ is a diagonal friction tensor and ξ(t) is a three-dimensional delta-correlated white noise $\langle \xi (t)\xi (t+\tau )\rangle \;=\;\delta (t,t+\tau )$. We integrated the Brownian dynamics using $\gamma =10^{-7}$ and a time step of $10^{-4}\;s$ over a 1 s duration using LAMMPS software (Release 2 August 2023—Update 3) [39]. The first half of the simulation was discarded as burn-in. Integrating the Brownian dynamics showed a reduced diffusion coefficient D of single chromatin beads for strong binding of C-type beads to B-type beads, and quasi-linear scaling of D with respect to temperature (Figure 4e). Therefore, we found that increased chromatin binding of C-type beads promotes a more compact chromatin polymer where individual beads diffuse more slowly due to crosslinking.

## 4. Discussion

Recent studies have demonstrated that BRD4 is present in discrete nuclear bodies that exhibit properties of other well-studied biomolecular condensates, including rapid recovery of fluorescence after photobleaching and sensitivity to 1,6-hexanediol. Both BRD4 long and short isoforms are found in phase-separated condensates in the nucleus and are associated with active gene transcription [27]. Importantly, CK2-mediated NPS phosphorylation regulates chromatin binding activity of BRD4 [32] as well as BRD4 phase separation [27]. This has led to the conclusion that phosphorylation of BRD4 inhibits interaction with chromatin and reduces phase separation, while remaining necessary for active gene transcription. Moreover, phosphorylated and unphosphorylated BRD4 form different molecular associations—transient polyvalent associations of unphosphorylated BRD4 contrast with the stable dimeric interaction and chromatin binding of phosphorylated BRD4 [31]. Our data support the model that BRD4 variants can form different molecular associations in the nucleus.

The experimental evidence presented here suggests that chromatin binding of BRD4 and phase separation are critical for maintaining NC structure and environment. Substantial colocalization of the 7D mutant with nucleosomes, along with increased chromatin compaction and decreased diffusion, points towards a BRD4-mediated crosslinking mechanism of nucleosomes within the NC. This result of increased affinity can also be seen in coarse-grained simulations of chromatin interactions with multivalent binders. The 7A mutant exhibits a less statistically significant localization to nucleosomes with respect to 7D and has no effect on NCs. However, reduced diffusion of nucleosomes in cells expressing the 7A mutant is consistent with increased phase separation ability of the 7A mutant. A similar trend was also observed for cells expressing the bromodomain-deactivated mutant (BD), where we have observed reduced nucleosome diffusion and no effect on the NCs.

Furthermore, modifications of the nucleosome microenvironment and clutch compaction are distinct between the BD mutant cells and cells exposed to (+)-JQ1. We found that the reduced diffusion of nucleosomes upon expression of the BD mutant to be a natural result due to loss of chromatin binding activity while maintaining intrinsically disordered regions compatible with the multivalent interaction capacity of BRD4. On the other hand, (+)-JQ1 is known to reduce chromatin binding activity [30] as well as condensation of BRD4 [27], which may lead to NC decompaction and increased diffusion. Lastly, reduced diffusivity of single nucleosomes for all mutants tested suggests a potential relationship between elevated colocalization and the degree of confinement of nucleosome diffusion.

These experimental findings were supplemented by representing chromatin as a string-of-beads model where multivalent particles, referred to as C-type beads, can bind to the chromatin backbone with varying affinity. The stochastic motion of a chromatin polymer and C-type beads in solution was simulated together via Brownian dynamics to observe the effects of C-type binding on the mobility of the polymer. As expected, we found that a multivalent binder, reminiscent of BRD4 condensates, can crosslink the polymer and constrain its motion. Similar results are seen experimentally, where modulation of BRD4 binding efficacy to chromatin alters both chromatin structure and dynamics. These results suggest a general and intricate role for BRD4 in the maintenance of chromatin nanodomain architecture.

We further hypothesize that nascent BRD4 condensates are seeded by promiscuous interactions of BRD4 with cofactors, followed by phosphorylation and chromatin interactions mediated by the phosphorylated form. The stable dimeric interaction and binding of phosphorylated BRD4 to acetylated chromatin would then mediate control of the chromatin architecture by promoting crosslinking of the chromatin fiber and acting as a molecular ‘bridge’ between transcriptional condensates with chromatin. Lastly, we hypothesize effects of BRD4 phase separation on the viscosity of the chromatin nanodomain environment. Indeed, we find this to be a natural result of molecular crowding resulting from overexpression of a constitutively phase-separating protein, capable of multivalent interactions.

## 5. Conclusions

Our findings highlight the role of BRD4 in nuclear condensate formation and its regulation of chromatin architecture and mobility. The distinct molecular associations formed by BRD4 mutants suggest a nuanced mechanism of chromatin recruitment, binding, and remodeling of the chromatin microenvironment. Further research should focus on the role of BRD4 phase separation in regulating chromatin structure, any dependencies of chromatin structure on BRD4 interaction with other nuclear cofactors, and downstream effects of phase separation on gene expression. Investigating how BRD4-mediated nucleosome crosslinking influences chromatin compaction, particularly in the context of molecular crowding and multivalent interactions, will provide deeper insights into its function.

## Data Availability

The raw algorithms for the colocalization analysis and the polymer simulations are available upon request.

## Supplementary Materials

The following supporting information can be downloaded at: https://www.mdpi.com/article/10.3390/cells15020118/s1, Figure S1: Density of FLAG puncta in a 10 μm box obtained by automated counting in different mutants. Error bars represent the standard error of the mean. No statistical significance is observed for any group. N = 10 cells per group; Figure S2: Ripley’s K analysis of FLAG-tagged BRD4 mutants. (left) Average K-function for FLAG-tagged BRD4 mutants and WT protein. (right) Individual K-functions to show variability in the number of FLAG tagged puncta surrounding H2B puncta. Dark lines indicate the mean, and regions show one standard deviation above and below the mean. Dashed lines indicate the theoretical K-function under completely spatial randomness (CSR) where $K\left(r\right)=\pi r^{2}$; Figure S3: Peak intensity and area of detected FLAG puncta (upper left) Peak intensity of FLAG puncta for BRD4 mutants and WT protein. (upper right) Area of FLAG puncta for BRD4 mutants and WT protein. (lower left) Histograms corresponding to data shown in upper left. (lower right) Histograms corresponding to data shown in upper right. N = 1000 per group; Figure S4: Uncropped gels for western blotting of BRD4 mutants. N = 2 biological replicates were conducted for this experiment.
