# Supplementary material for: BRD4 Phosphorylation Regulates the Structure of Chromatin Nanodomains
Source: Cells. 2026 Jan 9;15(2):118. doi: 10.3390/cells15020118 (PMC12838699; doi:10.3390/cells15020118)
Supplement: Supplementary file 1 [file cells-15-00118-s001.zip › cells-4045375-supplementary.pdf]

SUPPLEMENTARY INFORMATION

BRD4 Phosphorylation Regulates the Structure of Chromatin Nanodomains

Clayton Seitz [1,*], Donghong Fu [1], Mengyuan Liu [1], Hailan Ma [1] and Jing Liu [2,3,4,*]

[1] Department of Physics, Indiana University, Indianapolis, IN 46202, USA
[2] Department of Physics and Astronomy, Purdue University, West Lafayette, IN 47907, USA
[3] Purdue Institute for Cancer Research, Purdue University, West Lafayette, IN 47907, USA
[4] Melvin and Bren Simon Comprehensive Cancer Center, Indiana University, Indianapolis, IN 46202, USA
* Correspondence: seitzc@purdue.edu (C.S.); liu456@purdue.edu (J.L.)

Expression level of BRD4 mutants

To consider the expression level of various BRD4 mutants, performed density measurements of FLAG puncta and found no statistical significance in the density between different mutants (Supplementary Figure S1). Moreover, we have identified Ripley's K function as an additional measure which provides insights into the local density variations of FLAG-tagged BRD4 in our dataset. For each cell, we estimate $K(r)$, which is proportional to the number of FLAG localizations within a radius r of a given H2B puncta.

$$K(r) = \frac{a}{n(n-1)}\sum_{ij} I(d_{ij} \leq r)$$

where a represents the area of the window and n is the number of localizations. $I(d_{ij} \leq r)$ is an indicator function which is unity when the distance from the ith to the jth point is less than or equal to r and zero otherwise. For multi-type point patterns, as in the colocalization dataset, the ith localization is a H2B puncta and j^{th} is a FLAG puncta. The average $\langle K(r)\rangle$ is computed for each BRD4 variant over a range of length scales. As expected, we find that $\langle K(r)\rangle$ deviates significantly from complete spatial randomness and therefore H2B puncta and FLAG puncta colocalize. Second, we find no statistically significant difference in the K-function over any length scale for different mutants (Supplementary Figure S2 left). Indeed, over short length scales on the order of the nucleosome nanodomain size, FLAG puncta are equally abundant across all groups (Supplementary Figure S2, left inset). The agreement of the K-function between mutants over longer length scales further suggests that the overall density of different mutants is comparable between groups.

Measurement of the peak intensity of FLAG puncta shows no statistically significant difference of mutants with respect to WT (Supplementary Figure S3 left). The area of each FLAG puncta was measured by fitting a 2D Gaussian function to each spot. The σ values along the x and y directions can be used to calculate the 1σ area: $A_\sigma = 2\pi\sigma_x\sigma_y$. This is the area of the elliptical contour of the 2D Gaussian at one standard deviation along each axis and is independent of the Gaussian amplitude. We find that there is no statistical significance in the area of FLAG puncta between different groups (Supplementary Figure S3 right). Uncropped gels corresponding to Figure 1b can be found in Supplementary Figure S4.

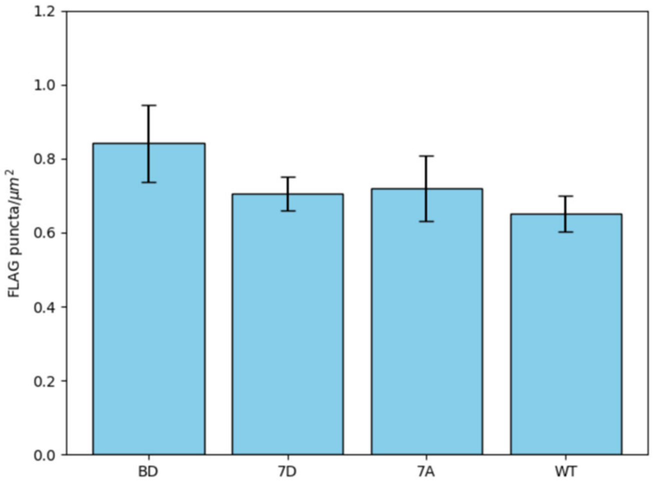

Supplementary Figure S1. Density of FLAG puncta in a 10 µm box obtained by automated counting in different mutants. Error bars represent the standard error of the mean. No statistical significance is observed for any group. N=10 cells per group.

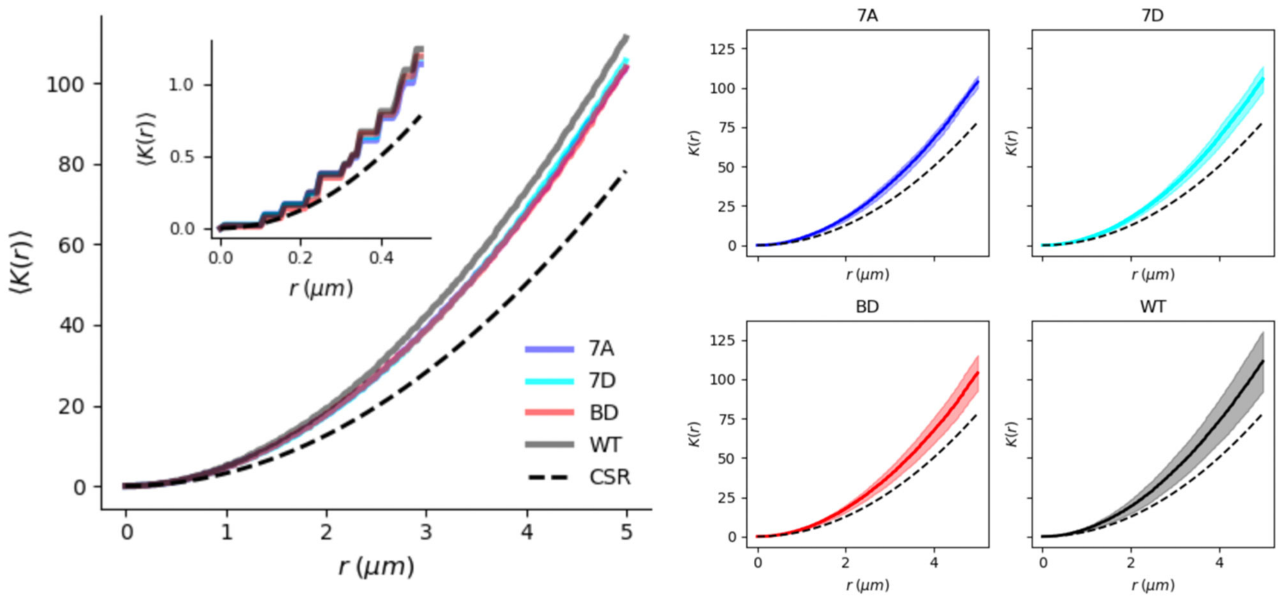

Supplementary Figure S2. Ripley's K analysis of FLAG-tagged BRD4 mutants. (left) Average K-function for FLAG-tagged BRD4 mutants and WT protein. (right) Individual K-functions to show variability in the number of FLAG tagged puncta surrounding H2B puncta. Dark lines indicate the mean, and regions show one standard deviation above and below the mean. Dashed lines indicate the theoretical K-function under completely spatial randomness (CSR) where $K(r) = \pi r^2$.

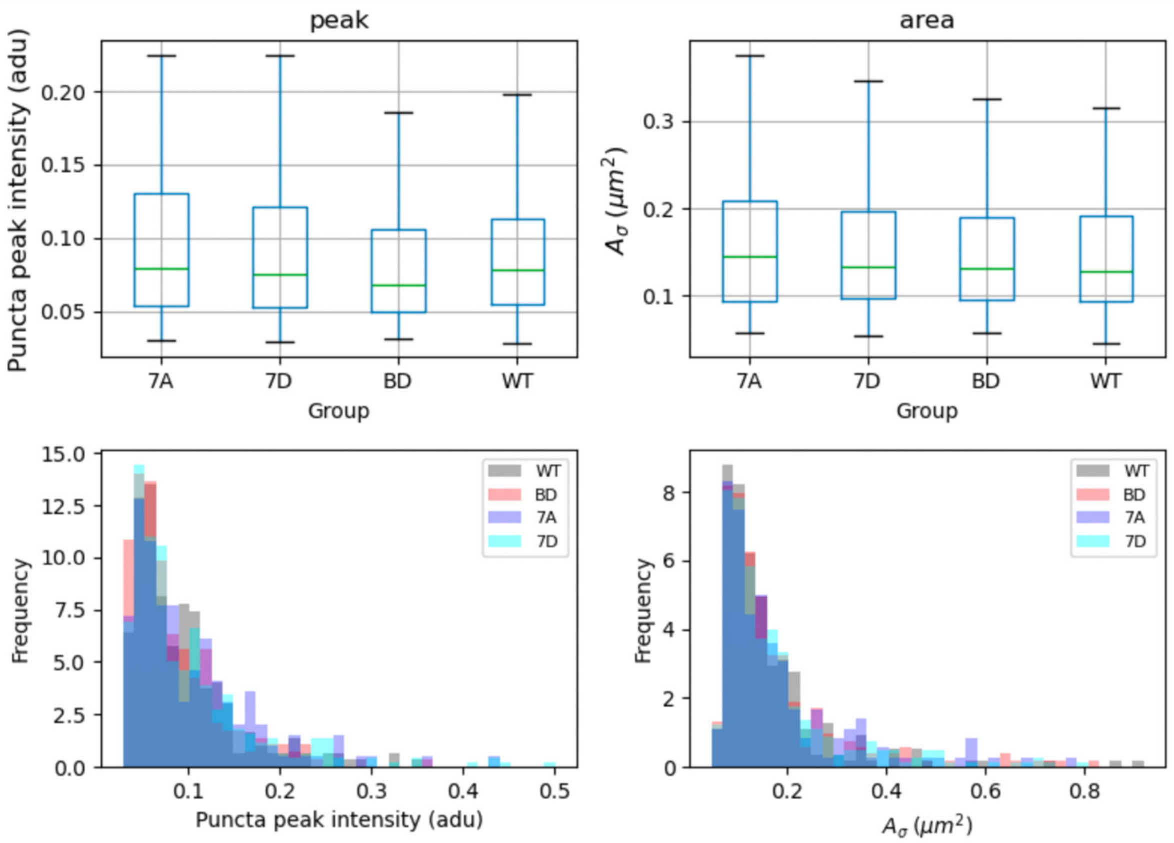

Supplementary Figure S3. Peak intensity and area of detected FLAG puncta (upper left) Peak intensity of FLAG puncta for BRD4 mutants and WT protein. (upper right) Area of FLAG puncta for BRD4 mutants and WT protein. (lower left) Histograms corresponding to data shown in upper left. (lower right) Histograms corresponding to data shown in upper right. N=1000 per group.

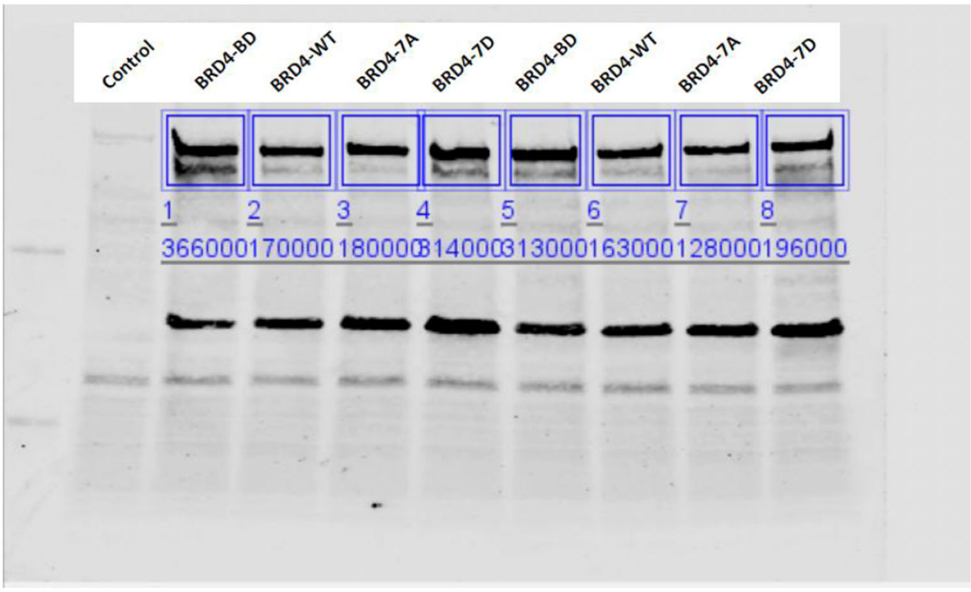

Supplementary Figure S4. Uncropped gels for western blotting of BRD4 mutants. N=2 biological replicates were conducted for this experiment.
